# From the archives: Somatic DNA demethylation, circadian rhythms LNKed to transcriptional machinery, and induction of Kranz anatomy in a submerged amphibious plant

**DOI:** 10.1093/plcell/koad016

**Published:** 2023-01-25

**Authors:** Louis-Valentin Méteignier

**Affiliations:** Assistant Features Editor, The Plant Cell, American Society of Plant Biologists, USA; PHIM Plant Health Institute, Univ Montpellier, INRAE, CIRAD, Institut Agro, IRD, Montpellier, France

## April 2022: Somatic DNA demethylation

Cytosine DNA methylation controls cell fate and stress responses by silencing transposable elements and regulating gene expression. The level of DNA methylation results from the balance between methyltransferase and demethylase activities. In Arabidopsis, active DNA demethylation is controlled by four DNA glycosylases/lyases: DEMETER (DME), REPRESSOR OF SILENCING1 (ROS1), DME-LIKE2 (DML2), and DML3. Heterozygous *dme/DME* plants produce only 50% of viable seeds without the *dme* mutation due to aborted embryos (Choi et al., 2002), precluding analysis of the role of *DME* in somatic tissues. To bypass this issue, **Ben P. Williams and colleagues** (Williams et al., 2022) created a somatic quadruple homozygous mutant of the entire DME family (called *drdd*). They constructed a *DME* transgene under the control of an embryo-specific promoter and introduced it into a triple mutant of ROS1, DML2, and DML3 (termed *rdd* mutant). They then crossed the transgenic homozygous *rdd/rdd* mutant with heterozygous *dme/DME* plants, and obtained transgenic wild type along with *dme*, *rdd*, *drdd* plants in which *DME* was expressed primarily in the embryo, thus rescuing the seed abortion phenotype of homozygous *dme* and *drdd* plants.

The authors analyzed leaf DNA methylomes and observed 276 differentially methylated regions in *dme*, 2,091 hypermethylated regions in *rdd,* and 2,601 hypermethylated regions in *drdd* compared to wild type, suggesting that the four demethylases function redundantly in leaves. By transcriptomic analysis, the scientists observed a correlation between gene hypermethylation and reduced transcript accumulation in *drdd*, suggesting that the DRDD pathway safeguards gene expression by “cleaning up” excessive DNA methylation. Among hypermethylated genes in *drdd*, the flowering time regulator *FLOWERING LOCUS C* was the most down-regulated gene, and *drdd* plants flowered earlier than the other genotypes. Importantly, natural Arabidopsis accessions with high DNA methylation at *FLOWERING LOCUS C* tended to flower earlier than low methylation accessions. By bypassing the embryo abortion phenotype of *dme*, the authors were able to explore how somatic DNA demethylation impacts flowering in Arabidopsis.

## April 2018: Circadian rhythms LNKed to transcriptional machinery

Biological systems possess circadian clocks that enable the coordination of cellular functions with diurnal light/dark cycles. In Arabidopsis, the regulatory component of the circadian clock is composed of numerous transcriptional regulators. Among them, the MYB transcription factor REVEILLE8 (RVE8) interacts with NIGHT LIGHT-INDUCIBLE AND CLOCK-REGULATED (LNK) proteins to regulate the transcription of dusk-expressed genes (Xie et al., 2014). Yuan Ma and co-workers (Ma et al., 2018) showed that the LHY-CCA1-LIKE (LCL) domain in RVE8 was required for the rhythmicity and amplitude of dusk gene expression, although a truncated form of RVE8 without LCL still interacted with dusk gene promoters through its MYB domain. To understand the DNA-binding-independent role of the LCL domain, they performed a genome-wide screen for LCL-interacting proteins and identified LNK proteins.

The authors then discovered that the enrichment of RNA polymerase II and a histone modification of active chromatin at dusk genes decreased in *lnk* plants. LNK physically interacted with RNA polymerase II and a histone chaperone subunit, and the association of RNA polymerase II and the histone chaperone with dusk-expressed genes decreased in *lnk* plants. The authors also showed that LNK and the histone chaperone are part of the same protein complex and that the chaperone is required for RNA polymerase II occupancy at dusk genes. Finally, the authors demonstrate that nascent RNA accumulation of dusk genes decreased in *lnk* mutant plants. This study provides a model for LCL action in RVE8 that is required for the interaction with LNK. In turn, LNK interacts with a histone chaperone complex. The results supported a role for this interaction in promoting the progression of RNA polymerase II along dusk-expressed genes.

## April 1998: Induction of Kranz anatomy in a submerged amphibious plant

Photosynthesis is the major player in atmospheric carbon fixation on earth and is essential for numerous heterotrophic organisms. C_3_ photosynthesis occurs in a single cell type: mesophyll cells. In contrast, C_4_ photosynthesis occurs in two distinct cell types: atmospheric CO_2_ is fixed in mesophyll cells as C_4_ acids that are then transferred to Kranz cells where they are decarboxylated to generate CO_2_ that will be re-fixed by ribulose-1,5-bis-phosphate carboxylase/oxygenase (Rubisco). The partitioning of C_4_ photosynthesis in two distinct cell types concentrates CO_2_ at the site of Rubisco activity and reduces competition for oxygen, hence favoring photosynthesis over photorespiration (Langdale, 2011). It is therefore crucial to understand the genetic regulation of C_3_ and C_4_ photosynthesis to envision the biological engineering of photosynthesis.

In general, plant species are either C_3_ or C_4_, but exceptions to the rule exist. The amphibious leafless sedge *Eleocharis vivipara* develops C_4_ anatomy and biochemistry when grown in terrestrial conditions, in contrast to C_3_ anatomy and biochemistry when grown in submerged conditions (see Figure). Twenty-five years ago, Osamu Ueno discovered that treating submerged *E. vivipara* plants with abscisic acid induced a shift from C_3_ to C_4_ anatomy and biochemistry (Ueno, 1998). The same research group recently discovered that gibberellic acid induces a shift from C_4_ to C_3_ anatomy, but not biochemistry, in terrestrial forms of *E. vivipara* (Suizu et al., 2021). The Ueno group studies suggest that transitions between C_3_ and C_4_ biochemistry are genetically uncoupled from anatomical transitions.

**Figure:**
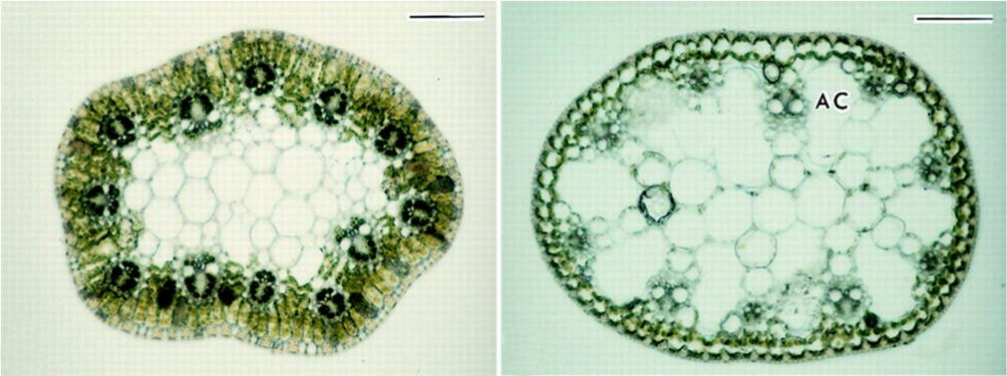
Cross-sections of culms of the terrestrial form (left) and submerged form (right) of *E. vivipara*. AC, air cavity, bars 100 μm (left), 50 μm (right). Adapted from Ueno (1998), Figure 1.
